# Magnetic Resonance Imaging Features of Congenital Infantile Fibrosarcoma

**DOI:** 10.7759/cureus.53132

**Published:** 2024-01-28

**Authors:** Ahmad AlQatie, Hatem Abbod, Tariq Alzaid, Afaf Alsolami

**Affiliations:** 1 Department of Radiology, King Faisal Specialist Hospital and Research Center, Riyadh, SAU; 2 Department of Radiology, Salmaniya Medical Complex, Manama, BHR; 3 Department of Pathology and Laboratory Medicine, King Faisal Specialist Hospital and Research Center, Riyadh, SAU; 4 Central Medical Laboratory and Blood Bank, Prince Sultan Military Medical City, Riyadh, SAU

**Keywords:** congenital mass, pathological features, imaging features, soft-tissue sarcoma, congenital infantile fibrosarcoma

## Abstract

Background

Congenital/infantile fibrosarcoma is a rare soft tissue tumor presented in early age of life. It should be considered in the differential diagnosis of the large soft tissue masses especially in the extremities at the age of infancy. These tumors frequently are misdiagnosed at birth as hemangioma. Histologically, they can resemble their adult counterparts and they are characterized by the chromosomal translocation t(12;15) (p13;q25) resulting in the *ETV6-NTRK3* gene fusion.

Objective

A retrospective review of the MRI features of histopathology-proven congenital/infantile fibrosarcoma provides our own institutional experience and supports the limited radiology literature written about this disease.

Material and method

The list of our patients is obtained after reviewing our radiology and pathology database in the period between June 1st, 2007 and May 31st, 2017 (10 years) at King Faisal Specialist Hospital & Research Center, Riyadh. Phrases used to search in our MRI examinations database are: congenital infantile fibrosarcoma, infantile fibrosarcoma, juvenile fibrosarcoma, soft tissue sarcoma, malignant soft tissue mass, sarcomatous soft tissue mass, fibrosarcoma, spindle cell sarcoma, myomatous sarcoma.

Result

In our database and picture archiving and communication system (PACS) during the period of the study, the word (fibrosarcoma) was mentioned in the radiology report of 182 patients. Only four cases were histopathologically proven to be a congenital/infantile fibrosarcoma and had completed their own MR exams - three of them were primary/new cases, males with an age range between 0 days and 5 months (median age: 5 months). The fourth case was a female with a history of 1^st^ presentation at the age of one month and proved by histopathology examination but there was no available imaging at that time; however, tumor recurrence in the same patient was at the age of 4 years with available MR imaging and pathology sample.

Conclusion

Congenital infantile fibrosarcoma is a rare entity that has no specific MRI findings. However, it should be always considered as part of the differential diagnosis of congenital soft tissue masses with aggressive behavior.

## Introduction

Congenital infantile fibrosarcoma is a rare tumor, constituting about 10% of soft tissue sarcoma in children [1]. It is reported as the most common soft tissue sarcoma in children below one year [2].

Grossly, these tumors are usually poorly circumscribed with grey-white or pale pink solid cut surface. Myxoid and cystic areas can be seen as well as areas of haemorrhage and necrosis. Microscopically, they are composed of solid sheets of uniform immature appearing fibroblasts or spindle cells with herringbone pattern with variable mitoses, cystically dilated spaces and vascular channels, haemorrhage, necrosis, and calcification. Scattered chronic inflammatory cells are characteristic of this entity. There is no specific immunohistochemical marker and most of the cases harbour the translocation t(12;15) (p13;q25) resulting in the ETV6-NTRK3 gene fusion.

Congenital fibrosarcoma is locally aggressive in nature but metastases are rare [2]. Metastases are more common when the tumors arise in the trunk rather than the extremities [3].

The purpose of this study is to retrospectively review the MR imaging features of the histopathology-proven congenital/infantile fibrosarcoma, provide our own institutional experience and support the limited radiology literature written about this disease.

## Materials and methods

Patients

Our list contains four patients diagnosed in the period between June 1st, 2007 and May 31st, 2017 (10 years).

Our inclusion criteria were: histologically confirmed congenital/infantile fibrosarcoma, cases with complete MRI study, and those below 14 years of age. Our exclusion criteria were: patients diagnosed by MR imaging with no available histopathology confirmation, patients with pathology confirmation but no available MRI examination, and patients' age above 14 years.

This study follows the ethical policies and guidelines of our institution in collecting the information of research.

Imaging review

A total MRI examination of four patients was performed utilizing Signa HDxt 1.5T (GE Healthcare, USA), MAGNETOM Avanto 3T (Siemens, Germany), MAGNETOM Trio 3T (Siemens, Germany) MRI machines. All examinations were obtained with tumor protocol including multiplanars and multisequentials MR images pre- and post-gadolinium.

All of the cases were radiologically reviewed by pediatric radiology consultants with three years of dedicated pediatric radiology fellowship training and experience of more than five years in pediatric radiology. In addition, all of these cases were pathologically reviewed by a soft tissue pathologist.

The data were collected and compiled in Microsoft Excel, version 2019 (Microsoft Corporation, Redmond, WA, USA). After checking for completeness and consistency, data were analyzed using IBM SPSS for Windows, version 26 (IBM Corp., Armonk, NY, USA).

Pathology review

Hematoxylin and eosin (H&E)-stained slides were reviewed by two soft tissue pathologists with the corresponding ancillary studies for each case including the immunohistochemistry and the cytogenetics findings.

## Results

Patients

The total number of patients is four. Three of them are males and presented as primary/new cases with an age range between 0 days and five months (median age is five months). One patient is a female and presented at the age of four years with a history of primary presentation at the age of one month. The patients' data are summarized in Table 1.

**Table 1:** The patient’s data

Case	Age	Sex	Clinical course of disease	Location	Mets
1	5 months	Male	Primary/new case	Right thigh	Suspected single metastatic right lung nodule
2	5 months	Male	Primary/new case	Left forearm	No metastasis
3	At birth	Male	Primary/new case	Right leg	No metastasis
4	4 years	Female	Recurrent	Right arm	Right axillary metastatic lymph nodes

Imaging features

**Figure 1:**
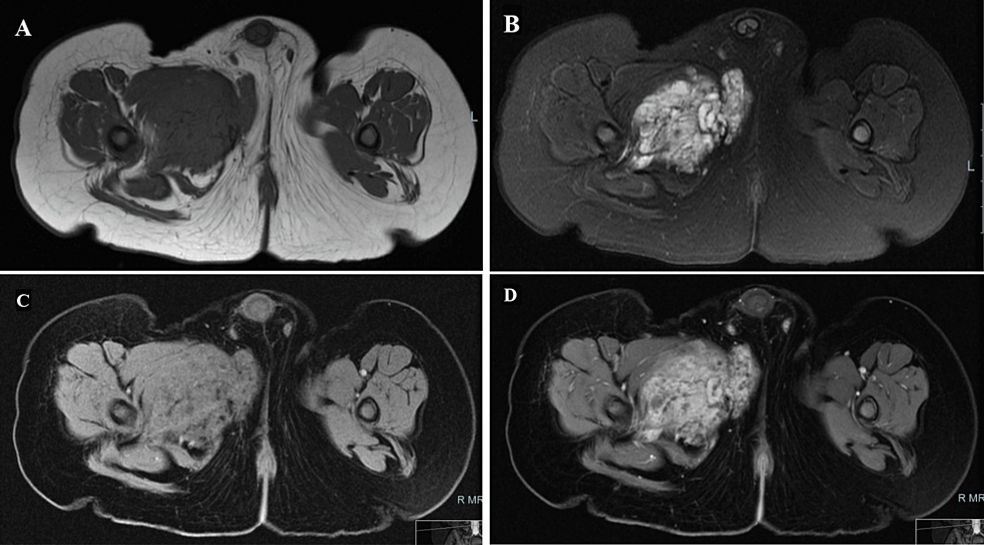
In Case 1, lobulated infiltrative soft tissue mass centered within the right upper medial thigh adductor muscles. (A) The tumor appears hypointense in axial T1WI. (B) Shows heterogeneous hyperintense signal in axial T2WI with FS. (C) Pre-gadolinium axial dry gradient image shows intermediate to low signal intensity of the mass. (D) Post-gadolinium axial image shows avid heterogeneous enhancement of the mass and demonstrates the extension of the lesion into the right side perineum. FS: Fat Suppression

The tumors in our four cases are located in the upper and lower extremities as follows: the right thigh, left forearm, right leg, and right arm. The tumors are variable in size with volume ranging from 72 cc to 672 cc (mean volume of the masses in the four cases is 243 cc). Ill-defined tumor margins and infiltrative behaviour are seen in all cases. Heterogeneous low signal intensity in T1WI and heterogeneous high signal intensity in T2WI with diffuse avid heterogeneous enhancement are seen in all four cases (Figure 1).

**Figure 2:**
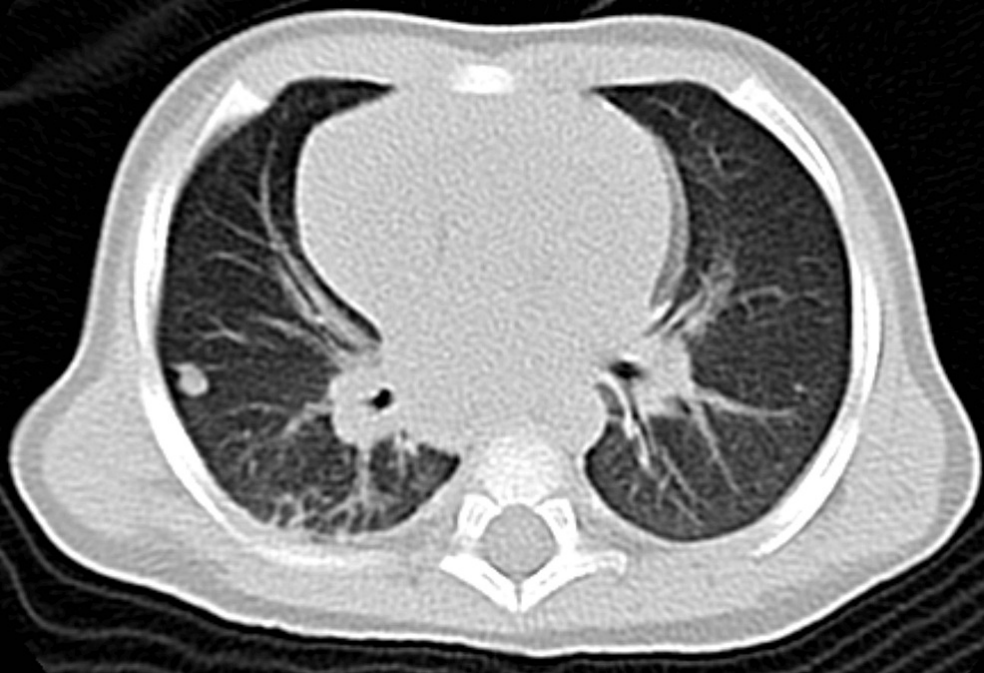
Single right lung nodule suggestive of metastases.

One case (25%) shows internal calcification. Another case (25%) shows patchy internal bleeding and areas of necrosis. The adjacent bone evaluation of the four cases reveals the following: intact bone in only one case (25%), mild periostitis with no infiltration in one case (25%), diffuse destruction of the adjacent bone seen in another case (25%), and ill-defined cortical bone erosion with no definite intramedullary extension seen in one case (25%). For metastases evaluation, metastases to draining lymph nodes are seen in one case (25%), and suspicion of a single metastatic lung nodule in another case (25%) (Figure 2).

Pathology features

The specimen types were as follows: in cases 1, 2 and 4 we received paraffin blocks and H&E stained slides representing large open biopsies/resections from other institutions while the remaining case (case No. 3) underwent an in-house biopsy.

Microscopically, a marked similarity between the cases is seen. The tumor cells are uniform oval to spindle with moderate atypia. They are packed in sheets with focal herringbone pattern. Scattered chronic inflammatory cells are noted and mitotic activity is variable. The background shows variable amounts of hemorrhage, hemosiderin deposition and necrosis (mainly in cases 1, 2 and 4). Also seen in the background are dilated vascular channels and in case 1 this was accompanied by prominent cystic changes with septations and calcifications (as seen in MRI); such changes can lead to the misdiagnosis of hemangioma.

In case 4, in addition to the first presentation at birth, the patient had a recurrence after four years. Both materials were available for evaluation. The recurrent tumor cells have the same morphology as described before. However, there was a pronounced nodular architecture and myxoid changes. This case was sent for an expert opinion who favored the diagnosis of recurrent infantile fibrosarcoma.

A panel of immunohistochemical studies was performed on every case including epithelial, neural, myogenic and vascular markers and all were negative.

**Figure 3:**
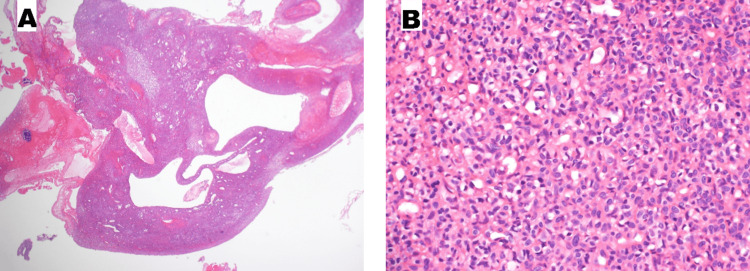
(A) In case 1, low power of the tumor shows cystic spaces and dilated blood vessels. An area of calcification, necrosis and haemorrhage is seen on the left side. (B) High power of the same tumor shows solid sheets of oval mesenchymal cells with moderate atypia and few mitoses.

The diagnosis was verified in cases 1 and 3 by Cytogenetics (FISH) study which showed ETV6 gene rearrangement (Figure 3).

## Discussion

Congenital infantile fibrosarcoma (CIFS) is a rare soft tissue tumor that can present at birth with the majority of patients presenting during the first year of life [4]. It has histopathological features similar to the adult type soft tissue fibrosarcoma but the infantile type of fibrosarcoma has better clinical behavior and better prognosis as compared to the adult type [5]. In our study, patients are presented early in life with a median age of five months. Males are affected more than females with a ratio of 3-4:1 (M:F) [1]. In our study, male to female ratio is 3:1. CIFS most frequently affects the extremities and less commonly affects the trunk, head and neck, mesentery, and retroperitoneum [4,6]. In our study, the tumor involved the extremities in all cases (100%). The risk of metastases is rare and accounts for 8% of tumors which are primarily located in the extremities. The risk of metastasis is increased to 26% if the primary tumor is located in the trunk [3,4]. In our study, only one patient (25%) has a suspicious single lung metastatic nodule and one patient (25%) has regional lymph node metastasis. Congenital infantile fibrosarcoma is known to have a risk for local recurrence. It has been shown to occur in 17-43% of patients after conservative surgery alone [4,7]. In our study, only one case (25%) developed recurrent local tumor after surgical removal with chemotherapy treatment. Despite MRI being the study of choice in the evaluation of soft tissue tumor, the radiological imaging of soft tissue tumor should be started by plain radiograph [8]. Plain radiograph can properly reveal osseous invasion by the soft tissue tumor, periosteal reaction and soft tissue calcification [8]. MRI is the best modality for the evaluation of soft tissue tumors because it has high image quality making easy detection of the lesion and helping in properly determining the boundaries of the mass, so it is considered as the most valuable imaging technique for preoperative anatomical staging [9]. MR imaging had a sensitivity of 93%, a high negative predictive value of 98%, and a specificity of 82% in identifying malignant lesions [10]. The MRI is needed to determine the extent of the mass, bone violation, neurovascular bundle involvement and joint affection which are important factors for local staging and treatment planning [11]. Another role of MRI is to determine the response of the soft tissue tumor to treatment [11]. The most commonly utilized MRI sequences in the evaluation of soft tissue tumor are spin echo, fat-suppressed images, and gradient echo sequences [11]. T1 weighted images are useful to detect the fat or haemorrhage within the tumor, T2 weighted images provide excellent contrast between the tumor and adjacent soft tissue [11]. MRI features of congenital infantile fibrosarcoma are isointense signal to muscle in T1WI, heterogeneous hyperintense signal in T2WI, heterogeneous enhancement in post gadolinium images, poorly defined tumor margins, scattered areas of low signal intensity in all sequences resulting from fibrous foci [3]. Fluorodeoxyglucose-positron emission tomography (FDG-PET) scan is being studied to assess the detection, grading and response to therapy of soft tissue sarcoma [12]. The MRI may help to differentiate benign from malignant masses [11]. The MRI features that suggest malignancy include large tumor size, irregular tumor margins, heterogeneous signal intensity of the tumor, neurovascular encasement and bone involvement [11]. CIFS prognosis is based on tumor size, local invasion, nodal spread and distant metastases [3]. Fibrosarcoma is the most common malignant soft tissue tumor in the age 0 to 5 years followed by rhabdomyosarcoma [3]. The most important differential diagnoses for CIFS are rhabdomyosarcoma, infantile myofibroma, and congenital hemangioma [2,4,5]. Rhabdomyosarcoma accounts for 10% to 15% of solid tumors in the extremities and 4% to 5% of childhood cancers [3]. Approximately two-thirds of rhabdomyosarcoma cases are diagnosed in children under the age of six years [11]. Infantile myofibroma may clinically resemble CIFS and it can present at birth [4]. The majority of cases are diagnosed within the first two years of life [4]. It usually presents as a subcutaneous nodule and may appear similar to hemangioma if it has sufficient vascularity [4]. Vascular malformation/congenital hemangioma is often mistaken for various infantile hypervascular tumor such as CIFS [2]. Hemangioma occurs in 12% of infants and by the age of five years, 50% are gone, and most regress by 10 to 12 years [3]. Hemangioma shows much more enhancement and appears more homogenous than CIFS [5]. Congenital infantile fibrosarcoma can also arise from the sacrococcygeal area and should be differentiated from the sacrococcygeal teratoma which mainly has benign MRI features [13].

The MRI is important to determine the best area to obtain a soft tissue biopsy which is needed to confirm the diagnosis [14]. The ETS variant gene 6-neurotrophin receptor 3 (ETV6-NTRK3) gene fusion product identified by RT-PCR is diagnostic for infantile fibrosarcoma and the ETV6-NTRK3 transcript was present in 87.2% of patients in the investigation performed by the European Pediatric Soft Tissue Sarcoma Study Group [15].

During clinical management of patients, close follow-up imaging is highly recommended due to the risk of local tumor recurrence [16]. Imaging every three months in the first year of treatment is suggested for congenital infantile fibrosarcoma masses originating in the extremity and trunk regions where the local tumor recurrent can be easily detected by clinical examination but for those congenital infantile fibrosarcoma masses that originate in a nonpalpable location such as in the retroperitoneal region, more frequent follow-up imaging is recommended [16].

The main treatment of congenital infantile fibrosarcoma in the extremity region is surgical excision [17]. Chemotherapy is given for large non-operable masses to decrease the tumor size before surgical resection and unfortunately, sometimes amputation is needed for a nonfunctional limp or if the tumor is not responding to the chemotherapy [17].

The prognosis of CIFS is good in comparison to adult fibrosarcoma with a higher probability of long-term survival (90% at 5 years) and with better response to chemotherapy treatment [18]. Taking into consideration, congenital infantile fibrosarcoma tumors are locally aggressive with a high incidence of tumor recurrence if the tumor is not properly resected with a good negative margin confirmed by histopathology examination [19].

This study has some limitations that should be considered. First, the retrospective design may have limited the completeness of data collection, as we were only able to review the imaging studies and not follow up with the patients to assess for any changes or progression in the findings.

## Conclusions

In conclusion, our study provides valuable information about congenital infantile fibrosarcoma and highlights the need for further research in this area. Congenital infantile fibrosarcoma should be considered in the differential diagnosis of soft tissue masses with MRI aggressive features such as hemorrhagic components, ill-defined borders, and central necrosis in infant patients. Correlation with histopathology result is mandatory for confirmation of suspected radiological diagnosis. MRI exam also has a good role in follow-up patients during and after treatment to assess response to treatment and to detect tumor recurrence early.
